# Pediatric brain tumor cells release exosomes with a miRNA repertoire that differs from exosomes secreted by normal cells

**DOI:** 10.18632/oncotarget.21621

**Published:** 2017-10-06

**Authors:** Ágota Tűzesi, Teresia Kling, Anna Wenger, Taral R. Lunavat, Su Chul Jang, Bertil Rydenhag, Jan Lötvall, Steven M. Pollard, Anna Danielsson, Helena Carén

**Affiliations:** ^1^ Sahlgrenska Cancer Center, Department of Pathology and Genetics, Institute of Biomedicine, Sahlgrenska Academy, University of Gothenburg, Gothenburg, Sweden; ^2^ Krefting Research Center, Department of Internal Medicine and Clinical Nutrition, University of Gothenburg, Gothenburg, Sweden; ^3^ Department of Clinical Neuroscience, Institute of Neuroscience and Physiology, Sahlgrenska Academy, University of Gothenburg, Gothenburg, Sweden; ^4^ MRC Centre for Regenerative Medicine, University of Edinburgh, Edinburgh BioQuarter, Edinburgh, UK; ^5^ Sahlgrenska Cancer Center, Department of Oncology, Institute of Clinical Sciences, Sahlgrenska Academy, University of Gothenburg, Gothenburg, Sweden

**Keywords:** microRNA, exosomes, cancer stem cells, glioma, pediatric

## Abstract

High-grade gliomas (HGGs) are very aggressive brain tumors with a cancer stem cell component. Cells, including cancer stem cells, release vesicles called exosomes which contain small non-coding RNAs such as microRNAs (miRNAs). These are thought to play an important role in cell-cell communication. However, we have limited knowledge of the types of exosomal miRNAs released by pediatric HGG stem cells; a prerequisite for exploring their potential roles in HGG biology. Here we isolated exosomes released by pediatric glioma stem cells (GSCs) and compared their repertoire of miRNAs to genetically normal neural stem cells (NSCs) exosomes, as well as their respective cellular miRNA content. Whereas cellular miRNAs are similar, we find that the exosomal miRNA profiles differ between normal and tumor cells, and identify several differentially expressed miRNAs. Of particular interest is miR-1290 and miR-1246, which have previously been linked to ‘stemness’ and invasion in other cancers. We demonstrate that GSC-secreted exosomes influence the gene expression of receiving NSCs, particularly targeting genes with a role in cell fate and tumorigenesis. Thus, our study shows that GSCs and NSCs have similar cellular miRNA profiles, yet differ significantly in the repertoire of exosomal miRNAs and these could influence malignant features of HGG.

## INTRODUCTION

Pediatric high grade gliomas (HGGs) are one of the most significant causes of morbidity and mortality among children due to their aggressive clinical behavior [1]. One of the driving forces behind the growth of HGG is thought to be a subpopulation of glioma stem cells (GSCs) [2]. The generation and maintenance of these tumorigenic cells is orchestrated in part by transcriptional and epigenetic changes [3], such as those induced by microRNAs (miRNAs).

MiRNAs are small non-coding RNAs (19-25 nucleotides long) and function as mRNA silencers and transcriptional regulators of gene expression. They have been identified to have important roles in tumor development and progression as oncomirs [3–5]. MiRNAs are also present in small (30-100 nm) sized membrane vesicles called exosomes [6, 7] which have an endocytic origin and are released by different cell types [8]. Exosomes have gained much attention since the discovery of their RNA content and the important role they have in cell to cell communication [9] as well as their possible role in tumor progression [10]. Previous studies have described miRNA expression in pediatric brain tumors [11, 12], differences between pediatric and adult gliomas [13], and in exosomes derived from adult glioblastoma (GBM) cell lines [14, 15]. However, nothing is known about the expression pattern of exosomal miRNAs, secreted by primary cell cultures originating from pediatric HGG patients. This is essential if we are to understand any potential role they have in the biology of HGG.

Here we used primary patient-derived GSCs to survey the expression of a large number of miRNAs – both exosome derived and cellular – in pediatric GSCs and compared these to normal neural fetal stem cells (NSCs) reference controls. We find that exosomes from GSCs altered the gene expression of recipient NSCs, suggesting that the exosomes could have a role in the tumor microenvironment influencing the properties of neighboring cells.

## RESULTS

### Characterization of exosomes released by glioma stem cells and neural stem cells

**Figure 1:**
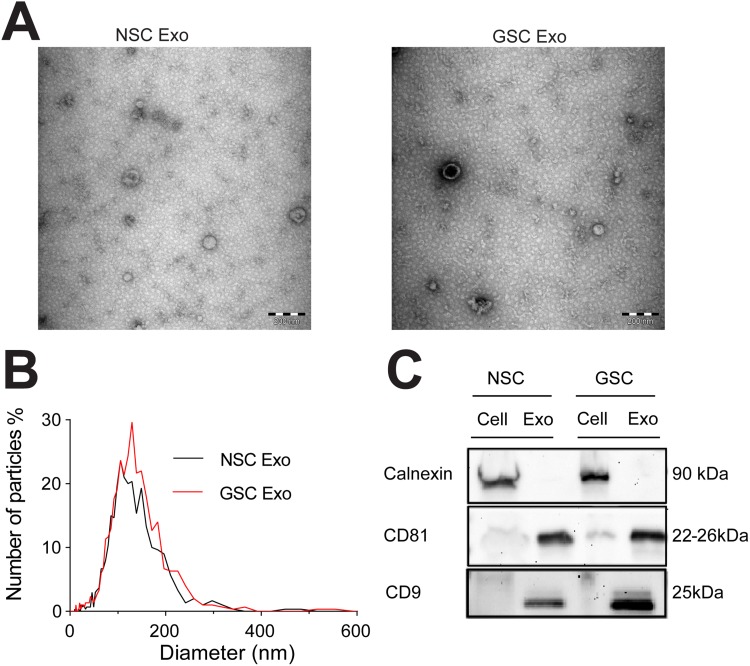
Characterization of exosomes isolated from neural and brain tumor stem cells **(A)** Representative image from transmission electron microscopy of exosomes (Exo) (for each sample group), scale bars: 200 nm. **(B)** Determination of exosome size distribution by Nanoparticle Tracking Analysis and **(C)** characterization with Western blot of exosomal surface markers CD9 and CD81, and cell-specific marker Calnexin in cells and exosomes.

We used NSCs and patient-derived primary cell cultures from pediatric HGG (GSCs) grown in stem cell conditions. The GSCs have previously been thoroughly described [16]. Briefly, the cells express stem cell proteins, respond to differentiation cues and are tumor-initiating when orthotopically injected into mouse brains. The NSCs also express stem cell markers and have the capacity to differentiate (Supplementary Figure 1 and [17]). Extracellular vesicles released by the cells were isolated from the cell culture media (see Material and Methods). We characterized the exosomes based on their size and morphology using Transmission Electron Microscopy and Nanoparticle Tracking Analysis. The analyses confirmed the typical exosome shape and size (Figure 1A–1B) [18]. Presence of the exosomal surface markers CD81 and CD9 was indicated by Western blot. By contrast, Calnexin, an endoplasmic reticulum marker only present in cells, was absent (Figure 1C) [18].

### The miRNA expression pattern differs in cells and exosomes

**Figure 2:**
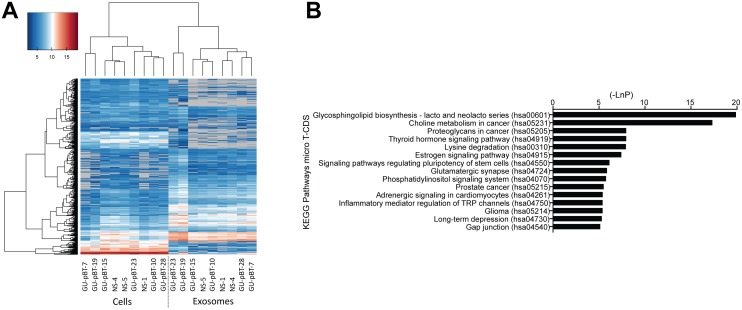
miRNA distribution and targeted pathways **(A)** Hierarchical clustering with Euclidean distance of log2 miRNA expression in all samples. Undetected miRNAs are colored grey. The exosomes and cells cluster separately based on their miRNA intensity. **(B)** Top 15 targeted KEGG pathways of the differentially expressed miRNAs between Glioma stem cell (GSC) exosomes and normal neural stem cell (NSC) exosomes include several relevant cancer pathways.

We analyzed miRNA expression in cells and exosomes from six GSC and three NSC lines and detected, in total, 1954 miRNAs. Among these, 250 miRNAs were present in both sets of samples (in both cells and exosomes); 480 were present in all cell samples; 389 were detected in all of the exosome samples. The detected miRNAs in exosomes (exosomal miRNAs) clustered separately from those in cells (cellular miRNAs) based on their expression level (intensity) (Figure 2A). We detected 334 miRNAs that were present only in cells (cell-specific miRNAs) and 355 miRNAs that were present only in exosomes (exosome-specific miRNAs). Hence, both the quantity and nature of the miRNAs differed between cells and exosomes based on expression levels and the type of miRNA expressed.

### Identification of differentially expressed miRNAs

We next focused on those miRNAs differentially expressed in the GSCs and NSCs. We detected 10 upregulated and 14 down-regulated miRNAs in the GSCs compared to the NSCs (Table 1, p<0.05). Reassuringly, the top up-regulated candidate was the glioma-associated miRNA-497-5p [19].

**Table 1:** Differentially expressed microRNAs in GSCs compared to NSCs

miRNA	FC^a^	*p*-value^b^	BH^c^
**Up-regulated**			
miR-497-5p	11.76	2.70E-06	1.30E-03
miR-551b-3p	3.04	5.92E-03	5.91E-01
miR-195-5p	2.99	5.79E-03	5.91E-01
miR-9-3p	2.68	2.08E-02	7.70E-01
miR-101-3p	2.48	2.95E-02	8.27E-01
miR-505-5p	2.26	4.90E-02	8.27E-01
miR-1199-5p	2.15	3.96E-02	8.27E-01
miR-487a-3p	2.09	2.67E-02	8.27E-01
miR-4783-5p	2.05	4.85E-02	8.27E-01
miR-187-5p	2.02	4.87E-02	8.27E-01
**Down-regulated**			
miR-29a-3p	2.20	4.77E-02	8.27E-01
miR-665	2.33	4.31E-02	8.27E-01
miR-4327	2.35	3.20E-02	8.27E-01
miR-3180	2.46	3.96E-02	8.27E-01
miR-4476	2.66	3.70E-02	8.27E-01
miR-3620-5p	2.59	1.72E-02	7.40E-01
miR-4688	2.72	1.85E-02	7.40E-01
miR-221-3p	5.61	1.84E-02	7.40E-01
miR-335-5p	6.12	1.75E-02	7.40E-01
miR-3131	3.19	1.15E-02	6.90E-01
miR-6880-5p	2.54	7.60E-03	5.91E-01
miR-4690-5p	2.82	8.63E-03	5.91E-01
miR-4486	3.08	6.88E-03	5.91E-01
miR-8089	3.86	4.23E-03	5.91E-01

*^a^*FC-Fold change, *^b^ p*-value <0.05 and *^c^* BH- Benjamini-Hochberg adjusted *p*-value.

We detected a larger number of differentially expressed miRNAs in the exosomes than in the cells; 37 miRNAs were up-regulated and five down-regulated in the GSC exosomes compared to the NSC exosomes (Table 2, p<0.05). Several of the upregulated miRNAs have not been identified previously in the context of GSC exosomes, such as miR-1290, miR-1246, miR-4299, miR-4732-5p, miR-6830-5p and miR-6165. We also detected many upregulated miRNAs that previously have been described to have a role in brain tumors such as miR-30c-1-3p [20], let-7a-5p [21], miR-24-3p [22] and miR-494-3p [23]. MiRNAs downregulated in the GSC exosomes included miR-4690-5p, miR-4443 and miR-198. We used qPCR to validate the miRNA array data, which confirmed the obtained results (Supplementary Figure 2). Enriched KEGG pathways targeted by the miRNAs that were differentially expressed between GSC and NSC exosomes included several cancer-related signaling pathways, such as Choline metabolism in cancer, Proteoglycans in cancer and Glioma (Figure 2B).

**Table 2:** Differentially expressed microRNAs in GSC exosomes compared to NSC exosomes

miRNA	FC^a^	*p*-value^b^	BH^c^
**Up-regulated**			
miR-4299	5.52	8.87E-05	1.38E-02
miR-4732-5p	5.49	9.23E-05	1.38E-02
miR-6830-5p	5.41	1.07E-04	1.38E-02
miR-7975	4.91	2.61E-04	2.34E-02
miR-1290	4.83	3.00E-04	2.34E-02
miR-1246	4.66	4.08E-04	2.65E-02
miR-3126-5p	4.34	7.49E-04	4.16E-02
miR-6715b-5p	4.27	8.64E-04	4.20E-02
miR-1273g-3p	4.09	1.22E-03	4.70E-02
miR-8078	4.02	1.39E-03	4.70E-02
miR-4428	4.02	1.40E-03	4.70E-02
miR-6165	3.89	1.81E-03	4.70E-02
miR-4454	3.75	2.40E-03	5.81E-02
miR-5100	3.70	2.65E-03	5.81E-02
miR-30c-1-3p	3.70	2.69E-03	5.81E-02
miR-652-5p	3.56	3.54E-03	7.25E-02
miR-4531	3.52	3.82E-03	7.43E-02
miR-513b-5p	3.30	6.06E-03	1.07E-01
miR-6778-5p	3.29	6.19E-03	1.07E-01
miR-6851-5p	3.28	6.31E-03	1.07E-01
miR-513a-5p	3.19	7.68E-03	1.24E-01
miR-4286	3.16	8.19E-03	1.27E-01
let-7a-5p	2.98	1.21E-02	1.74E-01
miR-5006-5p	2.96	1.27E-02	1.77E-01
miR-3162-5p	2.90	1.43E-02	1.86E-01
miR-4753-5p	2.89	1.47E-02	1.86E-01
miR-711	2.88	1.51E-02	1.86E-01
miR-665	2.87	1.53E-02	1.86E-01
miR-24-3p	2.83	1.69E-02	1.96E-01
miR-3714	2.82	1.72E-02	1.96E-01
miR-3122	2.75	2.00E-02	2.22E-01
miR-4649-5p	2.61	2.78E-02	3.01E-01
miR-6893-5p	2.57	3.03E-02	3.10E-01
miR-6870-5p	2.56	3.09E-02	3.10E-01
miR-7977	2.55	3.17E-02	3.10E-01
miR-494-3p	2.54	3.25E-02	3.10E-01
miR-4688	2.38	4.66E-02	4.32E-01
**Down-regulated**			
miR-4690-5p	3.91	1.75E-03	4.70E-02
miR-4443	3.91	1.74E-03	4.70E-02
miR-198	3.94	1.63E-03	4.70E-02
miR-3180	2.99	1.20E-02	1.74E-01
miR-6840-3p	2.53	3.27E-02	3.10E-01

*^a^*FC-Fold change, *^b^ p*-value <0.05 and *^c^* BH- Benjamini-Hochberg adjusted *p*-value.

In summary, we detected disease-associated pathways in the relevant miRNA expression differences between exosomes from GSCs and NSCs. Also, the exosome samples displayed a larger number of differentially expressed miRNAs between GSC and NSC than the cell samples, suggesting a specific active mechanism that is sorting miRNAs into exosomes and an important role of these vesicles in cell to cell communication.

### Identification of differentially expressed miRNAs in exosomes compared to cells suggest a potential role of the exosomal miRNAs

Next, we compared the miRNA expression of the exosomes to the expression in their originating cells and detected 152 differentially expressed miRNAs between NSCs and their exosomes, and 196 differentially expressed miRNAs between GSCs and their exosomes. Most of these miRNAs were up-regulated in the exosomes (NSCs: 100, GSCs: 147) (Supplementary Tables 1-2).

**Figure 3:**
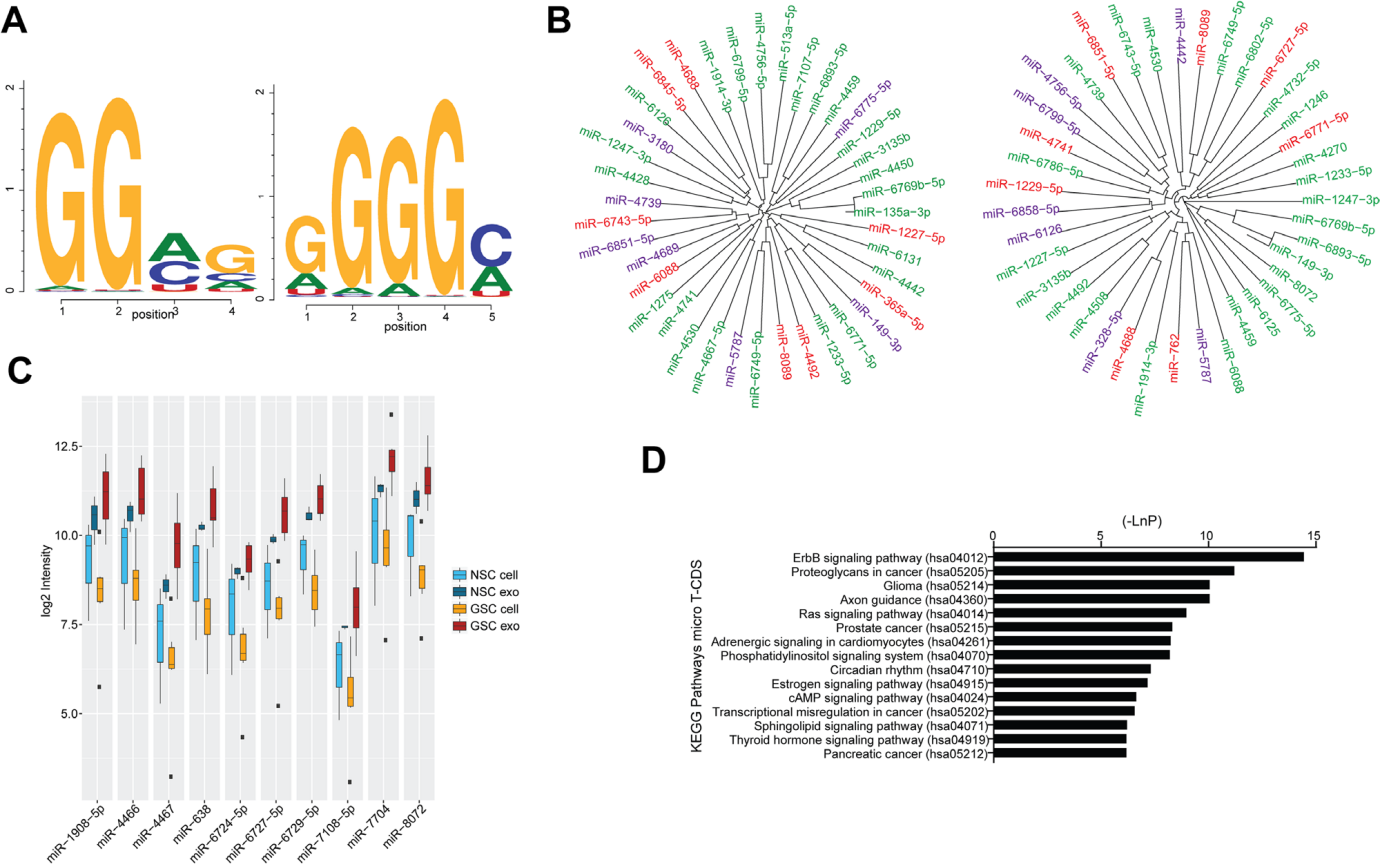
Exosomal miRNAs and their specific motifs **(A)** The two frequently present motifs in miRNAs overexpressed in Glioma stem cells (GSC) exosomes compared to GSCs and in normal neural stem cell (NSC) exosomes compared to NSCs. **(B)** miRNAs overexpressed in NSC exosomes compared to NSCs (left) and miRNAs overexpressed in GSC exosomes compared to GSCs (right) whose sequences contain GGAG (green), GGGGC (red) or both GGAG and GGGGC (purple). **(C)** Top 10 ‘glioma exosome specific’ miRNAs: these miRNAs have significant differential expression in the GSC exosomes compared to their cells while there is no significant differential expression in NSC exosomes compared to their originating cells and **(D)** the top 15 targeted KEGG pathways by the ‘glioma exosome specific’ miRNAs.

To determine why some of the miRNAs had higher intensities in the exosomes compared to their originating cells, we searched for motifs in their sequence. We detected two often repeating patterns (Figure 3A) in the miRNAs from the GSC and NSC exosomes. One of the sequence patterns, GGAG, has previously been described [24] and termed ‘exo motif’ and the second motif was GGGGC. Figure 3B displays miRNAs overexpressed in GSC or NSC exosomes compared to their cell counterparts and that contains one or both of the identified motifs.

Furthermore, we identified miRNAs that were differentially expressed between GSC exosomes and cells but not differentially expressed in NSC exosomes compared to their originating cells. This group of miRNAs, ‘glioma exosome specific’, was composed of 92 miRNAs, most of them (71) being up-regulated in the exosomes compared to their originating cells, for example miR-4467, miR-638 and miR-6727-5p (Figure 3C, Supplementary Table 3). In order to gain more information about the up-regulated ‘glioma exosome specific’ miRNAs, we searched for their targeted pathways using KEGG. Interestingly, most of the targeted pathways were cancer-, signaling- and glioma-related (Figure 3D). Based on these results, we hypothesize that loading of exosomal miRNAs are not random, but rather an effect of a well-defined mechanism.

### Exosomes from glioma stem cells alter the gene expression of recipient cells

**Figure 4:**
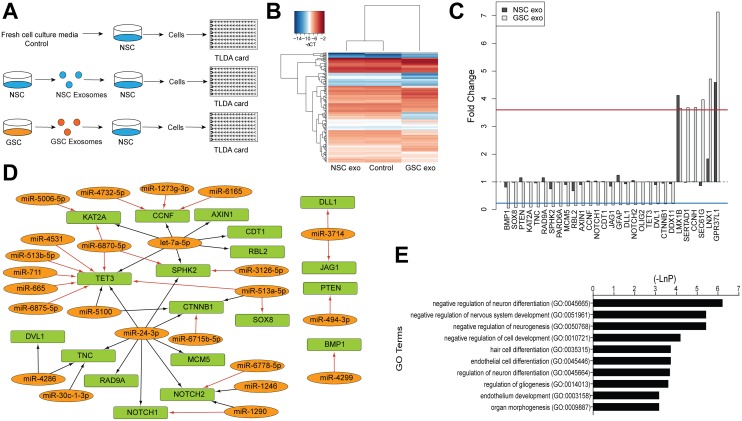
GSC exosomes effect on NSC gene expression **(A)** Experimental set-up. **(B)** Hierarchical clustering of log2 intensities of genes measured by the TLDA cards in the three different experimental conditions, displaying a different profile of the GSC exosome treated cells. **(C)** Gene expression in NSC after GSC exosome treatment, where fold change (FC) less than the blue line or above the red line was defined as differentially expressed (Methods). **(D)** Up-regulated GSC exosomal miRNAs (orange) and their target (validated, Tarbase, arrow in black and predicted, microT-CDS, arrow in red) mRNAs (green) that were downregulated in NSC after GSC exosomal treatment and **(E)** top 10 biological processes affected by the differentially expressed genes in the GSC exosome treated NSCs.

To determine if there is any potential functional role of exosomes secreted by GSCs, we aimed to test if the exosomes could affect normal recipient cells. We first verified, by confocal imaging, that exosomes can be taken up by NSCs (Supplementary Figure 3). Thereafter we treated cells with as follows, prior to gene expression analysis on TaqMan Low Density Array (TLDA) cards: 1) exosomes isolated from GSC (GU-pBT-28) media; 2) exosomes previously isolated from NSCs; and 3) no exosome treatment (Figure 4A). We profiled 192 genes that were predicted/validated target genes of the up-regulated GSC exosomal miRNAs or had a role in cell cycle, stemness, differentiation, glioma genesis, and neurogenesis.

We detected several gene expression changes induced by the GSC exosomes that were not induced by the NSC exosomes (Figure 4B). In total, 27 genes showed significant differential expression; 23 genes were down-regulated and four genes up-regulated (Figure 4C) in response to the GSC exosome treatment. Additionally, two up-regulated genes, *LMX1B* and *GPR37L1*, were also up-regulated in response to the treatment with NSC exosomes. Down-regulated transcripts included the tumor suppressor gene *PTEN* [25], stemness/differentiation/cell fate genes such as *NOTCH1*, *NOTCH2*, *JAG1*, *DLL1*, *GFAP*, and the cell cycle-related genes *RAD9A*, *MCM5* and *RBL2* (Figure 4C). The majority (19 of 23) of these down-regulated genes is predicted or validated mRNA targets for several of the up-regulated GSC exosomal miRNAs, which could indicate a direct effect (Figure 4D). For example, the GBM tumor suppressor gene *TET3* [26] is likely downregulated by the delivered exosomal miR-24-3p, known to be up-regulated in brain tumors [22] and validated to target *TET3* (Tarbase).

Up-regulated genes included the glioma related genes *SEC61G* and *LNX1* and the cell cycle related *SERTAD1* and *CCNH* (Figure 4C). The upregulation could be due to an indirect effect from the exosomal miRNAs or result from the mRNA content of the GSC exosomes.

Further, we examined which biological processes the altered genes are involved in and found relevant enriched GO terms such as nervous system development, neurogenesis or neuron differentiation (the genes measured by the TLDA cards was used as background for the enrichment analysis) (Figure 4E). In conclusion, these data suggest that GSC exosomal miRNAs altered the gene expression of the receiving NSCs in a manner that could affect several biological processes.

## DISCUSSION

Previous studies have catalogued the miRNA expression profiles of pediatric GBM tumors [12, 13]. However, for a better understanding of these tumors’ biology it is crucial to study the cancer stem cells which have an important role in cancer development, progression and recurrence [2]. These cancer stem cells are thought to be regulated by several epigenetic mechanisms, with miRNAs being one of them [3]. MiRNAs are present in exosomes, vesicles that play an important role in cell communication. Hence, we here determined miRNA profiles of pediatric HGG-derived cancer stem cells and their exosomes. We further investigated their effect on NSCs.

We detected few differentially expressed cellular miRNAs between the GSCs and the NSCs, but a larger number in the exosome samples. These miRNAs are validated or predicted to be involved in relevant signaling pathways suggesting that they could have a role in the tumorigenic process. The loading of the miRNAs with a higher expression in exosomes compared to the cells therefore does not seem to be random, but rather suggestive of an active mechanism of sorting/loading, at least partly based on the recognition of different miRNA motifs that are favored for loading into the exosomes [24]. The specific mechanisms underlying this observation require further investigation.

We identified several exosomal miRNA which have previously been reported to have a role in cancer and stemness [27]. These included two evolutionary young miRNAs, miR-1246 and miR-1290, that have been described to have a role in neuronal differentiation [28]. The secretion of these miRNAs through exosomes could potentially be a result of the GSCs attempting to maintain their stemness by eliminating factors that induce differentiation. Alternatively, as the same miRNAs have been associated with cancer cell proliferation, stemness, invasion and chemoresistance in several tumor forms [29–33], one could speculate that the tumor cells mediate tumorigenic signals to cells in their microenvironment through the exosomes. We therefore investigated the potential of the exosomes to alter recipient cells, by profiling gene expression changes in NSCs resulting from GSC exosome treatment, and detected down-regulated expression of glioma-associated tumor suppressors *PTEN* [34] and *TET3* [26]. Apart from inducing down-regulation of genes, the GSC exosomes also induced up-regulation of cancer-related genes such as *SERTAD1* and *SEC61G* [35–37] which would support the hypothesis that exosomes released by tumor cells can promote tumorigenesis. These changes were not a direct effect of the up-regulated GSC exosomal miRNAs, but rather an indirect effect of the miRNAs or explained by other material in the exosomes. One limitation of our study is the relatively limited number of samples included. However pediatric cancer stem cells are not easily obtained and our unique cultures represent one of very few collections of well-characterized cells that are currently available [16]. Future studies will be needed to extend the broader significance of our observations. Moreover the specific contribution of each miRNA identified here remains to be explored.

In conclusion, the present study describes the miRNA expression pattern in pediatric HGG-derived stem cells and in the exosomes derived therefrom, as well as their potential role in affecting recipient cells. The study provides new insights into epigenetic regulation of cancer stem cells from pediatric brain tumors, as well as the possible roles of the exosomes in the regulation of normal cells in their microenvironment. These findings may act as a basis for further studies towards better diagnostics or prognostic biomarker development, as well as for deciphering the specific role of exosomes in cancer stem cell maintenance, regulation and progression.

## MATERIALS AND METHODS

### Patients and samples

The study was approved by the regional ethics committee (Dnr 604-12). Tumor samples were obtained after signed informed consent from the parents of children who underwent surgery at the Sahlgrenska University Hospital, Gothenburg, Sweden.

### Cell cultures and exosome isolation

Established patient-derived primary cell cultures were grown in stem cell media (DMEM-F12 supplemented with B27 (Gibco), N2 (Gibco), EGF (20 ng/ml, Peprotech) and in some cases FGF-2 (20 ng/ml) on laminin (Sigma)-coated plastics as previously described [38]. Fresh media was added every 4 day. All established cell cultures were confirmed negative for mycoplasma contamination. The following cell lines were used: normal neural stem cells (NSCs): NS-1, NS-4, NS-5 and pediatric glioma stem cells (GSC): GU-pBT-7, GU-pBT-10, GU-pBT-15, GU-pBT-19, GU-pBT-23 and GU-pBT-28. The GSC lines are the same as in Wenger et al [16], described under different names, as follows: BPC-A7, BPC-B0, BPC-B5, BPC-B9, BPC-C3 and BPC-C8.

Conditioned medium was collected from each cell line and exosomes were purified by differential centrifugation [18]. In brief, the medium was centrifuged for 10 min at 300g to eliminate cell contamination. Supernatants were further centrifuged for 10 min at 2000g to eliminate apoptotic bodies, followed by ultracentrifugation with Ti70 fixed-angel rotor (Optima L-90 K Beckman Coulter). The supernatant was centrifuged at 28000x g (19400 rpm) in order to pellet microvesicles, followed by filtration of the supernatant through a 0.22 μm filter. Exosomes were pelleted by ultracentrifugation at 118000x g (40000 rpm) for 120 min. The exosome pellets were re-suspended in PBS.

### Exosome characterization

#### Western blot

For Western blot analysis, proteins were extracted from isolated exosomes with Radio-Immunoprecipitation Assay (RIPA) Buffer (Sigma-Aldrich) and volumes, corresponding to 20 μg of proteins, were separated on 10% a SDS-PAGE gel. Samples were then transferred onto a nitrocellulose membrane (Bio-Rad laboratories, Hercules, CA, USA), which was subsequently blocked in 5% Non-Fat Dry Milk (Bio-Rad Laboratories). The membrane was incubated with primary antibodies against Calnexin (1:1000; clone H-70; Santa Cruz Biotechnology, Santa Cruz, CA, USA), CD9 (1:1000, Santa Cruz Biotechnology, Santa Cruz, CA, USA) and CD81 (1:800; clone H-121; Santa Cruz Biotechnology, CA, USA) overnight at 4°C. Secondary antibody was ECL anti-rabbit IgG horseradish peroxidase-linked F(ab’)_2_ fragment (donkey-anti rabbit, 1:10000; GE Healthcare, UK). The membrane was visualized with ECL Prime Western Blotting Detection (GE Healthcare Life Sciences) and a VersaDoc 4000 MP (Bio-Rad Laboratories).

#### Nanoparticle tracking analysis

Size determination of exosomes was assayed using Zeta View (a Nanoparticle Tracking Analysis device: Particle Matrix, Germany).

#### Transmission electron microscopy

Exosomes (10μg) re-suspended in PBS were loaded onto Formvar/Carbon-coated grids (Ted Pella Inc., Redding, CA, USA) fixed in 2.5% glutaraldehyde, contrasted in 2% uranyl acetate and visualized with LEO 912AB Omega electron microscope (Carl Zeiss NTS, Jena, Germany) [39].

### RNA isolation and microarray

Total RNA from exosomes and cells were extracted using QIAzol Lysis Reagent (Qiagen). Glycogen (Invitrogen) was added to increase the yield of RNA. Extracted RNA from cells was treated with TURBO DNase (Invitrogen) and enriched in small RNA fraction with RNA Clean & Concentrator™-5 (Zymo Research). RNA was quantified with Qubit RNA HS Assay Kit (Invitrogen).

MiRNA microarray analyses were carried out with 3D-Gene Human miRNA Oligo chip ver.21 (Toray Industries), which detects 2565 miRNA transcripts. The intensity of each miRNA was analyzed with the 3D-Gene Scanner 3000 (Toray) with auto gain, auto focus and auto analysis settings, according to the manufacturer's instructions. The data normalization was performed on miRNA “spots” with background subtracted data. A normalization factor was calculated based on 25 divided with the median of the signal intensity of all background normalized data. Normalized data for each probe set and sample was calculated by multiplying background subtracted data with the normalization factor. The microarray data was analyzed with GenEx analysis software (MultiD Analyses).

### Quantitative real-time PCR (qRT-PCR)

The relative quantification of selected differentially expressed miRNAs was performed by qRT-PCR with the miRCURY LNA™ Universal RT microRNA PCR, Starter Kit with validated primer sets (Exiqon, Denmark) using ABI 7500 FAST Real-Time PCR System. Extracted total RNA from exosomes and small RNA enriched from cell samples were used for reverse transcription (20 ng) by adding UniSp6 RNA Spike-in template according to the manufacturer protocol. Since no stable endogenous control for exosomal miRNA exists, we used UniSp6 for normalization. ΔCt values were transformed to relative quantities and log2 calculation was applied in order to compare the qRT-PCR data with the microarray data. Pearson's Correlation was calculated between the qRT-PCR and microarray data for each sample group. All experiments were done in technical replicates.

### Immunocytochemistry

NS-5 was stained with mouse monoclonal nestin, R&D MAB1259, 1:500; rabbit polyclonal SOX2, Abcam ab97959, 1:1000; mouse monoclonal GFAP, Sigma-Aldrich G3893 and incubated overnight at 4°C. When applicable, EdU was visualized according to the manufacturer's instructions. Goat secondary antibody conjugated to Alexa dye, 1:1000 (Molecular Probes) was added for 1 h at room temperature and DAPI was used as a nuclear counterstain. Imaging of the cells was performed with the Operetta (Perkin Elmer). PKH67 Green Fluorescent Cell Linker for General Cell Membrane Labelling (Sigma-Aldrich) staining was used for exosomes for the confocal imaging (LSM 700 Carl Zeiss microscope) of the exosome up-take by cells.

### Functional study

Total RNA was isolated from NSCs treated daily for 8 days with 1) 30μg/ml exosomes from GSCs (GU-pBT-28), 2) NSC exosomes or 3) only media (no exosomes). cDNA was synthesized using the High-Capacity RNA-to-cDNA Kit (Applied Biosystems) and 15-50 ng cDNA was loaded per port on TaqMan Custom Arrays (Applied Biosystems) examining 192 genes in duplicates. The genes were possible target genes of the up-regulated GSC exosomal miRNAs or have roles in cell cycle, stemness, differentiation, glioma genesis, and neurogenesis. The arrays were run on a Viia7 Real-Time PCR system (Applied Biosystems) according to the manufacturer's instructions.

GAPDH was used as reference gene for ΔCT calculations, and hierarchical clustering was performed using Euclidean distance metric and average linkage. ΔΔCT-values were calculated between treatment 1) and 3) and treatment 2) and 3) (above), to estimate a Gaussian distribution. From this we estimated thresholds based on the mean ± 1.96^*^(standard deviation) to determine differentially expressed genes.

### Statistical methods

Differential expression was analyzed with *limma* in R [40], for miRNAs detected with the microarray in all samples included in the two groups of each comparison. Hierarchical clustering on miRNA expression data was performed on miRNAs detected in all cell line samples and/or all exosome samples, using Euclidean distance metric and average linkage.

The search for significantly over-represented motifs was done on miRNAs identified as overexpressed in exosomes compared to cells for GSCs and/or NSCs using the online application Improbizer (https://users.soe.ucsc.edu/~kent/improbizer/improbizer.html). As background we used the miRNAs included on the arrays that were not identified as overexpressed.

Clustering of miRNA sequences was done with ClustalW, default settings, using the R package *msa* [41], and visualization was done using the R package *ape* [42].

### Pathway analysis, Target gene prediction, network construction and GO analysis

Determination of targeted pathways based on Kyoto Encyclopedia of Genes and Genomes (KEGG pathways) for miRNAs was done using the DIANA TOOLS mirPath v3 software with the micro-T-CDS algorithm which predicts miRNA targets in CDS or 3’-UTR regions [43].

Based on Tarbase (validated miRNA targets) and microT-CDS (predicted miRNA targets) offered by the DIANA web tool, target networks were constructed for miRNAs and target genes with Cytoscape (Version 3.1.1) [44].

The involvement of target genes in biological processes was determined with the DAVID web tool using the GO BP_ALL settings [45, 46].

## SUPPLEMENTARY MATERIALS FIGURES AND TABLES









## Abbreviations

GBM: glioblastoma multiforme
HGG: high grade gliomas
GSC: glioma stem cells
NSC: neural stem cells
Exo: exosomes
KEGG: Kyoto Encyclopedia of Genes and Genomes
