# Supplementary material for: Pediatric brain tumor cells release exosomes with a miRNA repertoire that differs from exosomes secreted by normal cells
Source: Oncotarget. 2017 Oct 6;8(52):90164–75. doi: 10.18632/oncotarget.21621 (PMC5685739; doi:10.18632/oncotarget.21621)
Supplement: Supplementary file 1 [file oncotarget-08-90164-s001.pdf]

Pediatric brain tumor cells release exosomes with a miRNA repertoire that differs from exosomes secreted by normal cells

SUPPLEMENTARY MATERIALS

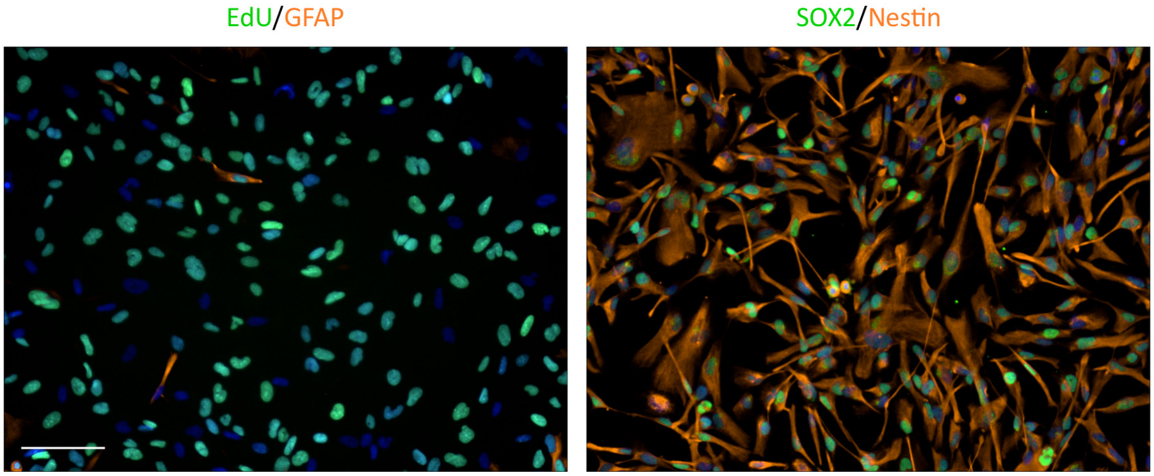

Supplementary Figure 1: Protein expression of NS-5. The normal neural stem cell line NS-5 is proliferative (visualized by EdU incorporation), has low expression of GFAP and is positive for the stem cell markers SOX2 and Nestin. The scale bar is 100 µm.

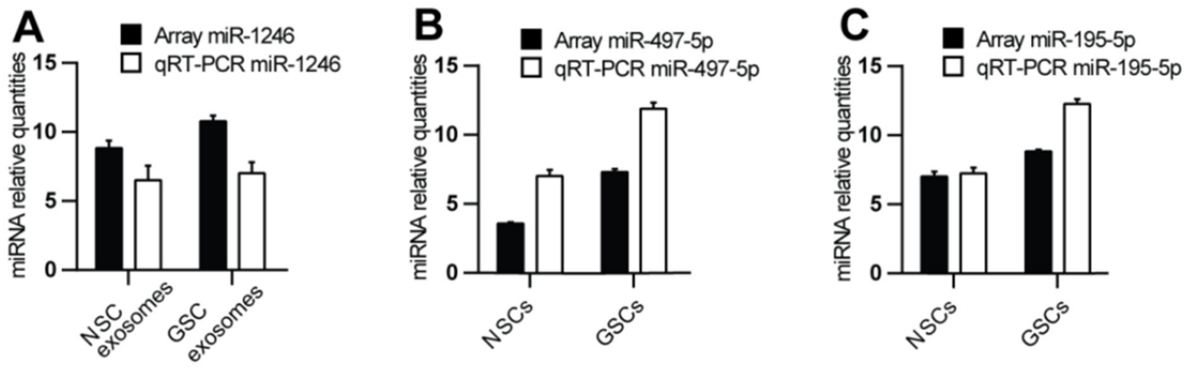

Supplementary Figure 2: Validation of selected miRNAs by qRT-PCR. Cellular and exosomal expression levels of selected miRNAs and Pearson correlation between microarray and qRT-PCR values for each sample group: miR-1246 (correlation: 0.90), miR-497-5p (correlation: 0.99), and miR-195-5p (correlation: 0.81). Error bars represent standard error.

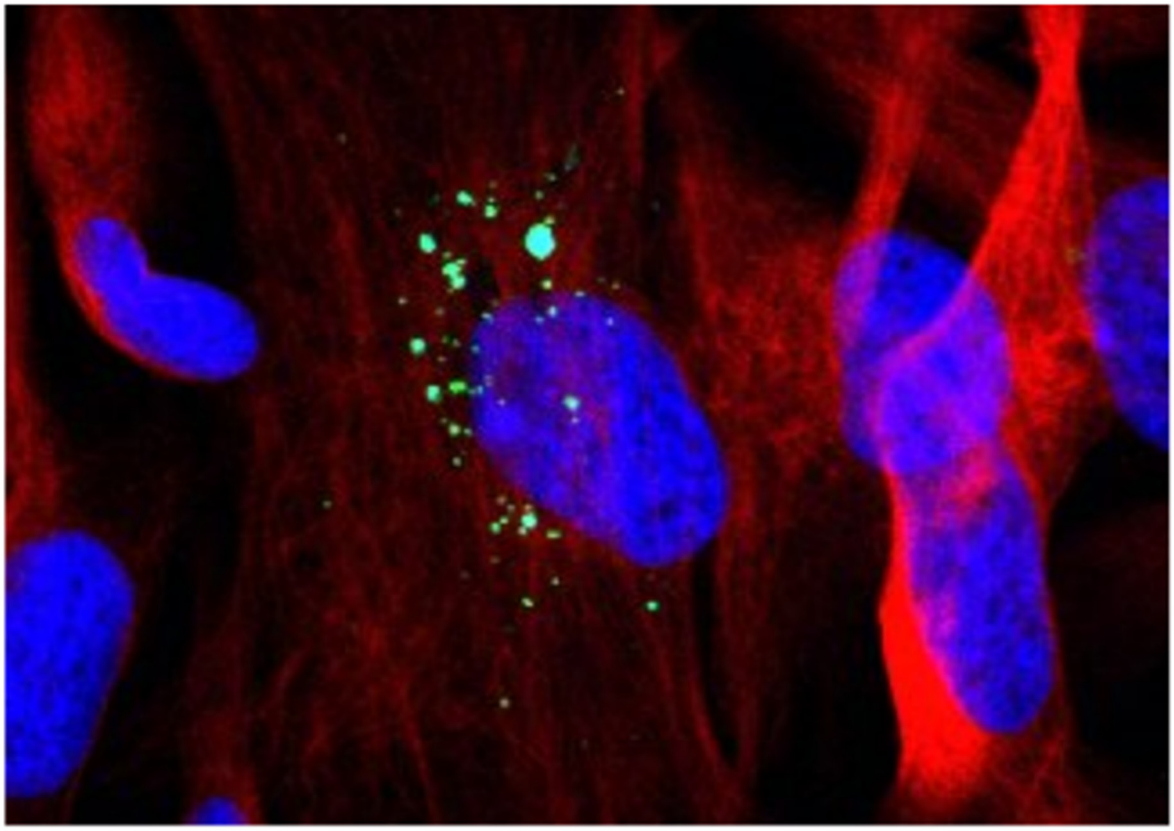

Supplementary Figure 3: Uptake of exosomes by NSC. Confocal image of the exosomal up-take by the NS-5 cell line stained with Nestin (red), Dapi (blue) and exosomes (green).

Supplementary Table 1: Differentially expressed miRNAs between normal neural stem cells (NSCs) and exosomes derived from NSCs

See Supplementary File 1

Supplementary Table 2: Differentially expressed miRNAs between Glioma stem cells (GSCs) and exosomes derived from GSCs

See Supplementary File 2

Supplementary Table 3: Glioma exosome specific miRNAs: miRNAs differentially expressed between glioma stem cells (GSCs) and GSC exosomes but not differentially expressed in NSC exosomes compared to NSC cells

See Supplementary File 3
